# Plasma phospholipids and fatty acid composition differ between liver biopsy-proven nonalcoholic fatty liver disease and healthy subjects

**DOI:** 10.1038/nutd.2016.27

**Published:** 2016-07-18

**Authors:** D W L Ma, B M Arendt, L M Hillyer, S K Fung, I McGilvray, M Guindi, J P Allard

**Affiliations:** 1 Department of Human Health and Nutritional Sciences, College of Biological Science, University of Guelph, Guelph, Ontario, Canada; 2 Toronto General Hospital, University Health Network, Toronto, Ontario, Canada; 3 Department of Medicine, University of Toronto, Toronto, Ontario, Canada; 4 Department of Pathology and Laboratory Medicine, Cedars-Sinai Medical Center, Los Angeles, CA, USA

## Abstract

**Background:**

There is growing evidence that nonalcoholic fatty liver disease (NAFLD) is associated with perturbations in liver lipid metabolism. Liver phospholipid and fatty acid composition have been shown to be altered in NAFLD. However, detailed profiles of circulating lipids in the pathogenesis of NAFLD are lacking.

**Objective:**

Therefore, the objective of the present study was to examine circulating lipids and potential mechanisms related to hepatic gene expression between liver biopsy-proven simple steatosis (SS), nonalcoholic steatohepatitis (NASH) and healthy subjects.

**Subjects:**

Plasma phospholipid and fatty acid composition were determined in 31 healthy living liver donors as healthy controls (HC), 26 patients with simple hepatic steatosis (SS) and 20 with progressive NASH. Hepatic gene expression was analyzed by Illumina microarray in a subset of 22 HC, 16 SS and 14 NASH.

**Results:**

Concentrations of phosphatidylethanolamine (PE) increased relative to disease progression, HC<SS<NASH (170<210<250 μg ml^−1^), and was significantly different (*P<*0.05) between HC and NASH. Circulating phosphatidylserine (PS) and phosphatidylinositol were higher in SS and NASH compared with HC (*P<*0.05), but there was no difference between SS and NASH. Fatty acid composition of phospholipids was also remodeled. In particular, docosahexaenoic and arachidonic acid were higher (*P<*0.05) in SS and NASH relative to HC in PS. Differentially expressed hepatic genes included *ETNK1* and *PLSCR1* that are involved in PE synthesis and PS transport, respectively.

**Conclusions:**

The present study demonstrates that there is a disruption in phospholipid metabolism that is present in SS, but more pronounced in NASH. Intervention studies targeted at lipid metabolism could benefit SS and NASH.

## Introduction

Obesity and the development of associated diseases such as cardiovascular disease, type 2 diabetes and nonalcoholic fatty liver disease (NAFLD) continue to be the leading causes of morbidity and mortality. Thus, identifying factors for the pathogenesis of obesity and associated diseases at an early stage are urgently needed.

NAFLD is a growing health problem, as it can lead to liver cirrhosis, end stage liver disease or hepatocellular carcinoma in some patients. Perturbations in phospholipid and fatty acid composition have been identified in mouse models and human patients with NAFLD,^1, 2, 3^ but more precise knowledge about perturbations in lipid metabolism could facilitate the development of effective new treatments for NAFLD. We have previously shown that the hepatic phosphatidylcholine (PC) to phosphatiylethanolamine (PE) ratio is decreased^4^ and that there are marked changes in essential fatty acid composition in the liver of NAFLD patients.^1, 5^ Altered PC/PE ratio was also observed in red blood cells,^4^ but this parameter was not measured in plasma. Plasma lipids under fasting conditions reflect mainly lipids and fatty acids exported from the liver, thus may also be potential biomarkers of altered liver lipid metabolism. Furthermore, a growing number of lipid classes have been identified in plasma that may have utility as biomarkers of disease risk. These include phosphatidylserine (PS), lyso-PC and sphingomyelin (SM), which have been associated with inflammation and cellular apoptosis.^6, 7, 8^ Therefore, the objective of the present study was to determine whether there are differences in circulating plasma phospholipid species between patients with simple hepatic steatosis (SS) or the progressive nonalcoholic steatohepatitis (NASH) and healthy living liver donors as healthy controls (HC). Correlations with hepatic gene expression pertaining to phospholipid metabolism were also examined to explore potential mechanisms of action.

## Materials and methods

### Patients

This was a cross-sectional study. Between October 2006 and February 2009, male and female ambulatory patients (age ⩾18 years) attending the liver clinic and healthy donors from the Living Donor Program for liver transplant recipients at the University Health Network, Toronto, Canada, were enrolled. From the liver clinic, we recruited patients with elevated liver enzymes (aspartate transaminase and/or alanine transaminase at least 1.5 times upper limit of normal) and suspected NAFLD undergoing diagnostic liver biopsy. Exclusion criteria: alcohol consumption >20 g d^−1^; liver disease of any etiology other than NAFLD (for example, viral hepatitis, autoimmune hepatitis and Wilson's disease); anticipated need for liver transplantation within 1 year or complications of liver disease; any reasons contraindicating a liver biopsy; medications known to precipitate steatohepatitis, supplementation of antioxidant vitamins, ursodeoxycholic acid or any experimental drug in the 6 months prior to entry; pregnancy or lactation. Controls were healthy living donors meeting the criteria as per transplantation protocol for the Living Donor Program. In addition, the same exclusion criteria as for NAFLD applied. Donors were recruited shortly before the planned transplantation. Each participant provided one fasting blood sample. Liver samples for histological assessment of NAFLD were obtained during a percutaneous needle biopsy for patients. Presence of a healthy liver in HC (no steatosis or cirrhosis) was confirmed by ultrasound, transient elastography, computed tomography and/or magnetic resonance imaging. In case of any doubt, a liver biopsy was performed. A wedge biopsy was taken for histology in all donors at the time of partial hepatectomy. Hepatic gene expression was measured in all groups and reported separately.^5^ The study was performed according to the guidelines of the 1975 Declaration of Helsinki and was approved by the Research Ethics Board, University Health Network, Toronto, Ontario, Canada (REB #03-0505-A, 8 October 2003, Amendments #2 for healthy donors, 29 August 2007). Approval was also obtained from the Research Ethics Board, University of Guelph, Guelph, Ontario, Canada (REB#10JN028, 9 July 2011). All subjects gave their informed written consent.

### Sample collection and preparation

Liver tissue was divided and stored in formalin for histological assessment, and in RNA preserving agent, RNAlater (Qiagen, Hilden, Germany), for gene expression analysis. Blood for phospholipid profiling was collected after overnight fast in ethylenediaminetetraacetic acid containing tubes and centrifuged (910* g*, 10 min). Plasma was collected and frozen at −80 °C until analysis.

### Liver histology

Two hematoxylin-eosin–stained sections for necroinflammatory grading and two Masson's trichrome–stained sections for assessment of fibrosis were reviewed. The Brunt system was used to determine the degree of steatosis, inflammation and fibrosis in NAFLD.^9^ Presence of steatosis was defined as having at least 5% of hepatocytes containing large-droplet fat. To distinguish between cases with SS and NASH, a separate category for zone 3 fibrosis characteristic of steatohepatitis as well as hepatocellular ballooning were evaluated. Lobular inflammation and NAFLD activity score were also assessed.^10^

### Plasma phospholipid analysis

Lipids from plasma were extracted according to the Folch method.^11^ Briefly, 100 μl of plasma was added to 4 ml chloroform:methanol solution (2:1, v/v). Samples were vortexed for  1 min, flushed with nitrogen gas and incubated at 4 °C overnight. The following day, samples were centrifuged at ~600 *g* for 10 min (21  °C) to separate phases. The lower chloroform layer was extracted and transferred to a fresh test tube, and dried under a gentle stream of nitrogen gas. The lipid was reconstituted in 100 μl of chloroform and spotted on a 2-cm scored lane of a 20 × 20-cm H-plate (VWR, Mississauga, ON, Canada, Silica Gel 60 pre-coated plates). The H-plate was previously activated by heating at 100 °C for 1 h. Phospholipid classes including SM, lyso-PC, PC, PS, phosphatidylinositol (PI) and phosphatidylethanolamine (PE) fractions were separated along with authentic standards in a solvent mixture of chloroform/methanol/2-propanol/KCl (0.25% w/v)/triethylamine (Fisher, Ottawa, ON, Canada and Sigma, Oakville, ON, USA) at a ratio of 30:9:25:6:18 (v/v). Bands were visualized under ultraviolet light after lightly spraying with 8-anilino-1-naphthalene sulfonic acid (0.1% w/v, Fluka, Oakville, ON, USA). Bands were scraped off the plate and transferred to a test tube containing a known amount of heptadecanoic acid (17:0). Lipids were converted to fatty acid methyl esters with the addition of 14% boron trifluoride in methanol (Sigma) and incubated at 100 °C for 1 h. Fatty acid methyl esters were quantified on an Agilent 6890 N gas chromatograph equipped with flame ionization detection (Mississauga, ON, Canada) and separated on an Supelco SP-2560 fused-silica capillary column (100 m, 0.2 μm film thickness, 0.25 mm internal diameter; Sigma). Samples were injected in splitless mode. The injector and detector ports were set at 250 °C. Fatty acid methyl esters were eluted using a temperature program set initially at 60 °C and held for 2 min, increased at 13 °C per min and held at 170 °C for 4 min, increased at 6.5 °C per min to 175  °C, increased at 2.6 °C per min to 185 °C, increased 1.3 °C per min to 190 °C, and finally increased 13 °C per min to 240 °C and held for 13 min. The run time per sample was 37.77 min. The carrier gas was hydrogen, set to a 30 ml per min constant flow rate. Peaks were identified by comparing with retention times of fatty acid methyl ester standards (Nu-Chek-Prep, Elysian, MN, USA) using EZchrom Elite version 3.2.1 software (Mississauga, ON, Canada). Quantitative amounts of phospholipid classes were calculated by proportional comparison of gas chromatography peak areas of total fatty acids with that of the 17:0 internal standard. Fatty acid percent composition was determined relative to total peak area.

### Analysis of hepatic gene expression by microarray

The analysis of hepatic gene expression has been described in detail previously^5^ and only genes related to phospholipid processes were examined for the present study. Briefly, total RNA from liver biopsies was extracted (mirVanaTM miRNA Isolation kit, Life Technologies Corp., Carlsbad, CA, USA), and RNA concentration, purity and quality were measured. Two hundred nanograms RNA were used for analysis with the Whole Genome Gene DASL HT Assay (Illumina Inc., San Diego, CA, USA) and the Human HT-12 V4 BeadChip (Illumina Inc.). Data were controlled for quality and analyzed using GeneSpring v12.5 (Agilent).

Differentially expressed genes (DEG) were identified by one way analysis of variance with a Benjamini–Hochberg false-discovery rate (FDR) q<0.05 and Tukey's HSD *post hoc* test. DEG were filtered for at least twofold up or downregulation. We searched the list of DEG among the groups for genes related to phospholipid metabolism. Expression levels (normalized but not median-centered and log2-transformed) for phospholipid-related DEG were then used for correlation analyses.

### Statistical analysis

**Figure 1:**
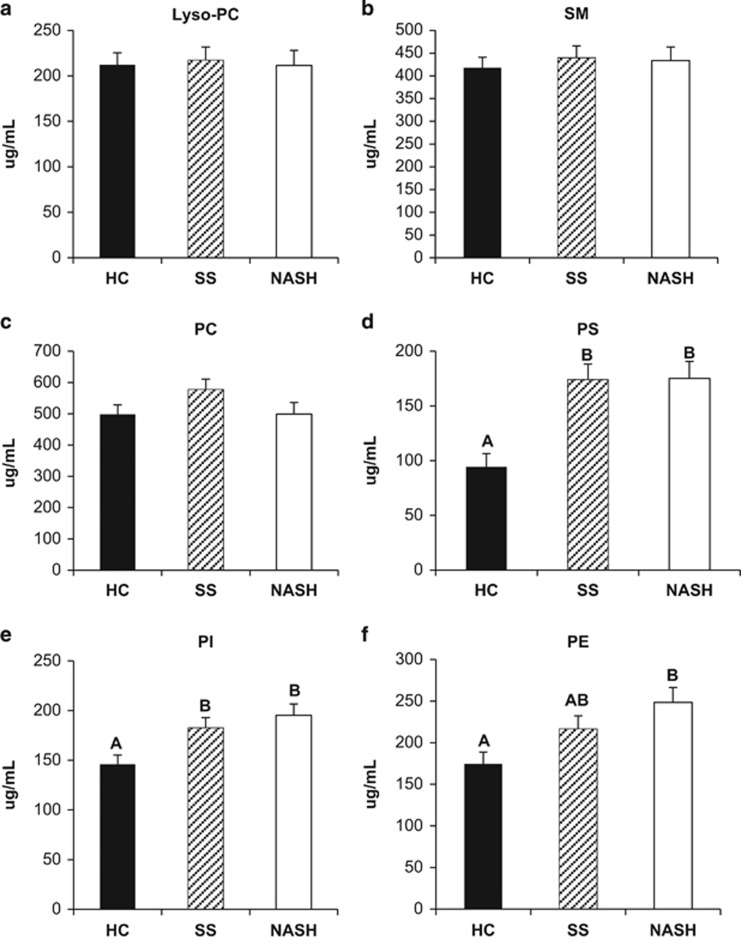
Lipid concentrations found in plasma phospholipid fractions (μg fatty acid  per ml of plasma) determined by gas chromatography for control (HC), SS and NASH subjects. (**a**) Lyso-phosphotidylcholine, *P*=0.95. (**b**) Sphingomyelin, *P*=0.81. (**c**) Phosphotidylcholine, *P*=0.08. (**d**) Phosphotidylserine, *P<*0.0001. (**e**) Phosphotidylinositol, *P*=0.0024. (**f**) Phosphotidylethanolamine, *P*=0.006. Bars represent means±s.d. (*n*=31 HC, 26 SS and 20 NASH). Bars not sharing a letter are significantly different (*P*<0.05) by Duncan's new multiple range test.

Data are presented as mean±s.d., median (25th; 75th percentile) or proportion of patients. The experimental data were subjected to a Wilcoxon test (continuous demographic and clinical data), Fisher's exact test (categorical data) or analysis of variance (phospholipid data) for discernment of statistical differences using a predetermined upper limit of probability of *P<*0.05. Multiple comparison testing of sample means was performed by the Duncan's multiple range test. The analysis was performed using the SAS system for Windows version 9.2 (SAS Institute Inc., Cary, NC, USA). The required sample size to detect a difference in means of 40 μg ml^−1^ (with a s.d. of 15 μg ml^−1^) in PI, the smallest fraction (Figure 1) at 90% power is *n*=9.

## Results

### Subjects

A total of 31 HC, 26 SS and 20 NASH plasma samples were analyzed. The SS group included three individuals who were recruited as healthy liver donors, but were found to have 5–10% steatosis on liver biopsy. Counting patients with a known diagnosis of diabetes or HbA1c ⩾0.065 or fasting glucose ⩾7.0 mmol l^−1^: HC: none, SS: 5/26 (19.2%), NASH: 5/20 (25%). All other patient and clinical characteristics for patients and controls are reported in Tables 1 and 2.

### Plasma phospholipids

Quantitative levels of plasma phospholipid species were determined from fasted blood samples. Plasma concentrations of PI and PS were higher in SS and NASH patients compared with HC (Figure 1), whereas PE was highest in NASH followed by SS and HC.

### Fatty acid composition of plasma phospholipids

The percent fatty acid composition was determined in each of the plasma phospholipid classes. The major n-6 and n-3 fatty acids are reported in Table 3. The essential fatty acids, linoleic acid and α-linolenic acid, and their long-chain polyunsaturated products arachidonic acid, eicosapentaenoic acid and docosahexaenoic acid (DHA) have an important role as substrates for the synthesis of pro and anti-inflammatory eicosanoids. Significant (*P<*0.05) differences for one or more of these fatty acids were found in all phospholipid classes. In particular, DHA was higher in SS and/or NASH patients relative to HC in several phospholipid species, with the largest differences (threefold and fivefold increase in SS and NASH, respectively) in PI and PS. Arachidonic acid was also higher in SS and NASH patients relative to HC in PS. In contrast, arachidonic acid in PE was lower in NASH than in HC and SS. Differences were also observed for linoleic acid in lyso-PC (lower in SS and NASH than in HC) and SM (higher in NASH than in HC). The changes in single fatty acids also lead to altered n3/n6 ratios in all phospholipid classes.

### Hepatic gene expression

Among the patients (31 HC, 26 SS and 20 NASH) that provided plasma for analysis, 52 (22 HC, 16 SS and 14 NASH) of these study participants also provided tissue for hepatic gene expression analysis as part of our previous study.^5^ There was no statistically significant difference between these patients and those who did not have gene expression measured (diagnosis, demography and clinical data) except for fasting glucose, which was slightly higher in those who had gene expression assessed (median (25th; 75th percentile)); (5.2 (4.7; 5.8) versus 4.9 (4.5; 5.2) mmol l^−1^; *P=*0.0358). Microarray data are presented in Table 4. Several genes related to phospholipid metabolism were differentially expressed between HC, SS and NASH in current participants. Correlations between these genes and plasma phospholipids are shown in Table 4.

## Discussion

We have shown that specific plasma phospholipids differ between HC, SS and NASH. We also found that these changes are associated with changes in the expression of genes involved in phospholipid metabolism in the liver. These findings suggest that in NAFLD, there is a disruption in phospholipid metabolism that is already present in SS, but more pronounced in NASH compared with HC.

We showed previously that liver PC/PE ratio, a biomarker of NAFLD, was lower in patients with SS and NASH compared with HC, and there was no difference between SS and NASH.^4^ In the present study, we show PC was not different, but plasma PE differed slightly among patient groups that was lowest in HC and higher in NASH (Figure 1). Although there was a trend toward lower plasma PC/PE ratio, where HC>SS>NAHS, there was no difference among groups. These observations suggest that compensatory mechanisms act to maintain circulating plasma PC/PE ratio at the expense of lower liver phospholipids.^12^ This compensation has been observed in mice lacking the gene for PE methyl transferase, required for the synthesis of PC in the liver and lipoprotein transport.^13^ Thus, although the liver is able to compensate for impairments in phospholipid synthesis at early stages of the disease, this may not be sustainable for the long term leading to observable differences in circulating PE, observed in the more advanced stage of NAFLD, that is, NASH.

There is growing recognition that plasma contains a multitude of lipids that may have utility as biomarkers of chronic disease. In addition to PC and PE, SM, lyso-PC, PS and PI were also determined in HC and NAFLD patients. Although these species are not as well understood as PC and PE, their presence may also have important health implications. These phospholipid classes are associated with inflammation and cellular apoptosis, and therefore may be associated with NAFLD and disease severity (SS versus NASH).^7, 8, 14, 15, 16, 17, 18^ PS was significantly higher (*P<*0.05, Figure 1) in SS and NASH as compared with HC, but no difference was detected between SS and NASH. PS has an important role in cellular apoptosis. Typically, it is found in the inner leaflet of the plasma membrane, but is externalized to the cell surface in response to stimuli signaling for cell death.^6^ We hypothesized that it could be higher in NASH versus SS, but this was not significant. In tandem, the fatty acid composition of PS was observed to have significantly higher levels of arachidonic and DHA in SS and NASH patients, relative to HC (Table 4). Arachidonic acid is liberated by phospholipase A2 for the production of eicosanoids that is involved in inflammatory processes.^19^ In contrast, DHA is known for its anti-inflammatory properties. Reasons for the presence of higher levels of these opposing fatty acids in PS are unclear. PI was also higher in SS and NASH patients relative to HC. PI and related metabolites are important second messengers involved in mitogen activated protein kinase and protein kinase B (PKB/Akt) signaling pathways.^20^ Although low levels of PS and PI have been reported in plasma, their significance in circulation are not entirely clear, but may influence the structure and function of lipoproteins.^21, 22, 23, 24^ No differences were found in lyso-PC and SM among HC, SS and NASH.

The observed changes in plasma phospholipids of PE, PS and PI in SS and NASH patients suggest alterations in phospholipid metabolism by the liver. Indeed, plasma lipids have been shown to be potential biomarkers of lipid composition in the liver and other tissues. PC containing DHA has been shown to be a marker of liver PE N-methyltransferase in human liver.^25^ The omega-3 index, derived from the sum of eicosapentaenoic acid and DHA from total lipids or phospholipids of red blood cells is a marker of these fatty acids in heart tissue and cardiovascular health.^26, 27^ Changes in fat intake in men have been associated with significant changes in plasma SM, ceramides, ether phospholipids, plasmalogens and gangliosides, and elevations in PC and PI.^28^ Alcoholic liver cirrhosis was shown to be correlated with plasma, cholesterol, lyso-PC, PC and PI.^29^

Corresponding to changes in circulating plasma phospholipid classes, analyses of liver gene expression identified DEGs related to phospholipid metabolism that were significantly (>twofold) over or underexpressed in NASH and SS relative to HC patients or between NASH and SS (Table 4). Search of PE-related genes identified ethanolamine kinase (ETNK1), which was downregulated in NASH and SS patients. ETNK1 is involved in the first step in PE synthesis, and recently identified as a gene involved in squamous cell carcinoma and gastric cancers.^30, 31^ PI-specific genes differentially expressed included lower expression of plasminogen activator urokinase receptor (PLAUR, transcript variants 1 and 2), a glycosyl-phosphatidylinositol (GPI)-anchored protein, and higher expression of myotubularin-related protein 4 (MTMR4) that is involved in the PI cycle and may influence plasma cholesterol.^32^ The PS-related gene, scramblase 1 (PLSCR1) responsible for phospholipid membrane ‘flip-flop', and maintaining membrane asymmetry and potentially for the movement of intracellular PS to the extracellular face during apoptosis was lower in SS and NASH.^33^ The expression of acyl-CoA-binding protein gene (DBI) involved in PC metabolism was higher in SS and NASH.^34^ ENPP2 or autotaxin, a lysophospholipase D enzyme involved in the production of lysophosphatidic acid (LPA) has been implicated in inflammation, and cancer was more highly expressed in NASH than SS.^35^ These changes in gene expression provide potential new avenues for further investigation to delineate how phospholipid metabolism is involved in the development of NAFLD.

A strength of the present study is both quantitative and qualitative measurements of lipid classes and fatty acids in tandem. In addition, analyses of liver samples from true HC are not readily accessible. The cross-sectional nature of the present study is a limitation that does not allow us to determine whether perturbations in lipid metabolism are causal or a consequence of NAFLD. This could be elucidated in prospective cohort studies with repeated measurements of lipids in patients at risk of developing NAFLD.

In summary, the present study provides evidence that the plasma phospholipid profile of NAFLD patients differ from HC, and that these changes are already present in SS and more pronounced in NASH. This suggests that there are changes early in the disease and that interventions targeting improvement in lipid metabolism may benefit both SS and NASH. Further intervention studies are warranted to assess this.

## Figures and Tables

**Table 1:** HC, SS and NASH patient obesity and hepatic steatosis

*Characteristic*	*HC*	*SS*	*NASH*	P*-value*
	*Percentage of patients (*n*/*N*)*	*Percentage of patients (*n*/*N*)*	*Percentage of patients (*n*/*N*)*	
*Obesity status*				*P<*0.05
Normal weight	41.9 (13/31)	26.9 (7/26)	15.0 (3/20)	
Overweight	45.2 (14/31)	46.2 (12/26)	20.0 (4/20)	
Obesity	12.9 (4/31)	26.9 (7/26)	65.0 (13/20)	
				
*Gender*				NS (*P*=0.10)
Female	51.6 (16/31)	26.9 (7/26)	55.0 (11/20)	
Male	48.4 (15/31)	73.1 (19/26)	45.0 (9/20)	
				
*Steatosis grading*^a^				*P<*0.05
0	100 (31/31)	0.00 (0/26)	0.0 (0/20)	
1	0.0 (0/31)	65.4 (17/26)	30.0 (6/20)	
2	0.0 (0/31)	19.2 (5/26)	35.0 (7/20)	
3	0.0 (0/31)	15.4 (4/26)	35.0 (7/20)	

Abbreviations: 0, <5% steatosis in % of hepatocytes involved; 1, 5–33% steatosis; 2, 33–66% steatosis; 3, >66% steatosis; HC, healthy controls; NASH, nonalcoholic steatohepatitis; NS, not significant; SS, simple steatosis.

a Steatosis grading according to Brunt *et al.*^9^

**Table 2:** HC, SS and NASH clinical characteristics

	*HC*			*SS*			*NASH*		
	N	*Median*	*25–75th*	N	*Median*	*25–75th*	N	*Median*	*25–75th*
Age (years)	31	38.0	18.0	26	45.5	12.0	20	41.5	17.5
Cigarettes (*n* per day)	31	0.0	0.0	26	0.0	0.0	20	0.00	0.0
Alcohol (g d^−1^)	31	0.0	4.0	26	0.3	2.0	20	0.9	4.0
Steatosis (% of hepatocytes)	15	0.00	0.00	26	17.50	30.00	20	42.50	42.50
NAS score	13	0.00	0.00	25	1.00	1.00	20	5.00	1.50
BMI (kg m^−2^)	31	25.61	6.24	26	27.87	5.19	20	32.67	7.18
Waist (cm)	30	87.75	15.66	24	97.57	12.00	19	106.90	20.75
AST (U l^−1^)	31	19.00	7.00	26	30.50	8.00	20	50.00	26.50
ALT (U l^−1^)	31	16.00	9.00	26	51.50	27.00	20	84.00	62.50
ALP (U l^−1^)	31	62.00	15.00	26	72.00	29.00	20	75.00	36.50
Glucose (mmol l^−1^)	31	4.90	0.60	24	5.10	1.10	18	5.25	1.50
Insulin (pmol l^−1^)	24	21.00	24.00	24	59.00	65.00	17	108.00	90.00
HOMA-IR	24	0.81	0.78	23	2.71	3.01	17	5.08	4.06
HbA1c	31	0.054	0.004	22	0.054	0.003	18	0.057	0.008
C-peptide (pmol l^−1^)	23	537.00	317.00	24	854.50	514.00	17	1318.00	233.00
Total cholesterol (mmol l^−1^)	24	4.80	1.51	25	4.90	1.28	18	5.30	1.08
Triglyceride (mmol l^−1^)	24	0.91	0.53	25	1.31	0.87	18	1.77	1.13
LDL (mmol l^−1^)	23	3.01	0.91	24	3.07	1.33	17	2.93	1.00
HDL (mmol l^−1^)	23	1.27	0.52	24	1.13	0.42	18	1.11	0.36
Bilirubin (μmol l^−1^)	29	10.00	7.00	25	10.00	4.00	20	10.00	8.50

Abbreviations: ALP, alkaline phosphatase; ALT, alanine transaminase; AST, aspartate transaminase; BMI, body mass index; HbA1c, hemoglobin A1c; HDL, high-density lipoprotein cholesterol; HOMA-IR, homeostasis model of assessment for insulin resistance; LDL, low-density lipoprotein cholesterol; NAS, nonalcoholic fatty liver disease activity score.

**Table 3:** Plasma phospholipid fatty acid composition

	*Lyso-PC*			P*-value*	*SM*			P*-value*
	*HC*	*SS*	*NASH*		*HC*	*SS*	*NASH*	
18:2n6	14.7±0.6^A^	12.3±0.7^B^	11.8±0.7^B^	<0.01	0.9±0.3^B^	1.6±0.3^AB^	2.1±0.4^A^	<0.01
18:3n3	ND	ND	ND	ND	ND	ND	ND	ND
20:4n6	3.0±0.2	3.4±0.2	2.9±0.3	0.78	1.1±0.9	1.3±0.2	1.3±0.2	0.59
20:5n3	0.1±0.1	0.1±0.1	0.3±0.1	0.14	0.04±0.04	0.1±0.04	0.01±0.04	0.08
22:6n3	0.2±0.1^B^	0.5±0.1^A^	0.2±0.1^B^	<0.01	N.D.	0.1±0.03	0.1±0.03	0.11
n3/n6	0.2±0.1^B^	0.6±0.1^A^	0.5± 0.1^A^	0.03	0.1±0.05^B^	0.2±0.05^A^	0.1±0.0^A^	0.04

	*PC*			P*-value*	*PS*			P-*value*
	*HC*	*SS*	*NASH*		*HC*	*SS*	*NASH*	
18:2n6	23.5±0.6	21.5±0.7	22.0±0.7	0.07	9.4±0.7	10.3±0.8	11.9±0.9	0.10
18:3n3	0.3±0.02	0.3±0.02	0.3±0.03	0.77	ND	ND	ND	ND
20:4n6	10.6±0.3	10.4±0.4	9.4±0.4	0.06	9.4±0.9^B^	16.5±1.0^A^	15.2±1.1^A^	<0.01
20:5n3	0.8±0.1^B^	1.1±0.1^A^	0.9±0.1^AB^	0.03	0.9±0.2	1.2±0.3	1.1±0.3	0.64
22:6n3	2.6±0.2^B^	3.5±0.2^A^	2.7±0.2^B^	<0.01	1.7±0.4^B^	5.9±0.5^A^	5.2±0.5^A^	<0.01
n3/n6	0.12±0.01^B^	0.16±0.01^A^	0.14±0.01^AB^	<0.01	0.14±0.02^B^	0.27±0.03^A^	0.24±0.03^A^	<0.01

	*PI*			P*-value*	*PE*			P*-value*
	*HC*	*SS*	*NASH*		*HC*	*SS*	*NASH*	
18:2n6	6.4±0.3	6.7±0.3	7.3±0.3	0.16	8.9±0.5	8.7±0.5	9.5±0.6	0.62
18:3n3	0.1±0.1	0.01±0.1	ND	0.70	0.1±0.04	0.1±0.04	0.1±0.1	0.51
20:4n6	20.7±0.6	21.8±0.7	20.1±0.7	0.18	27.0±1.0^A^	27.0±1.1^A^	25.0±1.2^B^	0.04
20:5n3	0.1±0.04	ND	0.1±0.1	0.26	1.4±0.02^B^	2.4±0.5^A^	2.0±0.02^AB^	<0.01
22:6n3	0.2±0.1^A^	0.6±0.1^B^	1.0±0.2^B^	0.01	7.2±0.5^AB^	9.7±0.5^A^	8.3±0.6^B^	<0.01
n3/n6	0.02±0.01^B^	0.03±0.01^B^	0.05±0.01^A^	0.03	0.09±0.03^B^	0.27±0.04^A^	0.15±0.04^B^	<0.01

Abbreviations: lyso-PC, lyso-phosphatidylcholine; ND, not detected; PE, phosphatidylethanolamine; PI, phosphatidylinositol; PS; phosphatidylserine; SM, sphingomyelin.

Percent fatty acid composition in plasma phospholipid fractions for healthy controls (HC), simple steatosis (SS) and nonalcoholic steatohepatitis (NASH) subjects. Values are expressed as mean±s.d. (*n*=31 HC, 26 SS and 20 NASH). Within a row and phospholipid fraction, values not sharing a superscript letter (A,B) are statistically different (*P*<0.05) by Duncan's new multiple range test.

**Table 4:** Hepatic gene expression by microarray and the correlation of differentially expressed genes in HC, SS and NASH patients for genes with known phospholipid associated function

*Probe ID*	*Entrez gene ID*	*Fold change*			P*-value*	*Symbol*	*Synonyms*
		*NASH vs HC*	*SS vs HC*	*NASH vs SS*			
*PC*							
840678	5168	1.43	−1.47	2.10	6.87E−05	ENPP2	NPP2; ATX; LysoPLD; ATX-X; PDNP2; PD-IALPHA; FLJ26803
2480338	1622	2.03	2.25	NS	6.11E−07	DBI	MGC70414; ACBD1; ACBP
							
*PE*							
2600224	55 500	−2.58	−2.18	NS	1.78E−10	ETNK1	EKI1; EKI; Nbla10396
							
*PS*							
3890609	5359	−1.91	−2.15	NS	7.23E−05	PLSCR1	MMTRA1B
							
*PI*							
360475	5329	−2.09	−2.27	NS	5.58E−07	PLAUR	CD87; UPAR; URKR
2120068	9110	2.05	2.16	NS	4.60E−09	MTMR4	FYVE-DSP2; ZFYVE11; KIAA0647

Abbreviations: HC, healthy controls; NASH, nonalcoholic steatohepatitis; NS, not significant; PC, phosphatidylcholine; PE, phosphatidylethanolamine; PI, phosphatidylinositol; PS; phosphatidylserine; SS, simple steatosis.

Genes were selected on the basis of their association with specific phospholipid fractions. Then, correlations were determined among HC, SS and NASH patients.
